# Extensive epigenomic integration of the glucocorticoid response in primary human monocytes and *in vitro* derived macrophages

**DOI:** 10.1038/s41598-019-39395-9

**Published:** 2019-02-26

**Authors:** Cheng Wang, Luca Nanni, Boris Novakovic, Wout Megchelenbrink, Tatyana Kuznetsova, Hendrik G. Stunnenberg, Stefano Ceri, Colin Logie

**Affiliations:** 1 Department of Molecular Biology, Radboud Institute for Molecular Life Sciences, Faculty of Science Radboud University, PO box 9101, 6500 HG Nijmegen, The Netherlands; 2 Department of Electronics, Information and Bioengineering (DEIB), Politecnico di Milano, Piazza Leonardo da Vinci 32, 20133 Milano, Italy; 3 Present Address: Murdoch Childrens Research Institute, Royal Children’s Hospital, Parkville, Australia; 4 Present Address: Department of Medical Biochemistry, Academic Medical Centre of the University of Amsterdam, Amsterdam, The Netherlands

## Abstract

Glucocorticoid receptor is a transcription factor that is ubiquitously expressed. Glucocorticoids are circadian steroids that regulate a wide range of bodily functions, including immunity. Here we report that synthetic glucocorticoids affect 1035 mRNAs in isolated healthy human blood monocytes but only 165 in the respective six day-old monocyte-derived macrophages. The majority of the glucocorticoid response in monocytes concerns genes that are dynamic upon monocyte to macrophage differentiation, whereby macrophage-like mRNA levels are often reached in monocytes within four hours of treatment. Concomitantly, over 5000 chromosomal H3K27ac regions undergo remodelling, of which 60% involve increased H3K27ac signal. We find that chromosomal glucocorticoid receptor binding sites correlate with positive but not with negative local epigenomic effects. To investigate further we assigned our data to topologically associating domains (TADs). This shows that about 10% of macrophage TADs harbour at least one GR binding site and that half of all the glucocorticoid-induced H3K27ac regions are confined to these TADs. Our analyses are therefore consistent with the notion that TADs naturally accommodate information from sets of distal glucocorticoid response elements.

## Introduction

Glucocorticoids are essential circadian steroid hormones that regulate peri-natal development^1^, emotion processing and memory^2,3^ the immune system^4^ and metabolism^5,6^. Synthetic glucocorticoids display potent immune suppressive properties^7,8^ and are used to treat various haematopoietic disorders and a wide range of inflammatory and autoimmune conditions. In the case of purified primary human monocytes (Mo) and *in vitro* monocyte-derived macrophages (Mf), glucocorticoids promote a tolerogenic state^9^ that has been called the ‘M2c’ polarisation state^10^. Similarly, dendritic cell maturation to a pro-inflammatory state is negatively affected by glucocorticoid treatment^11^. Glucocorticoids increase phagocytosis of myelin, bacteria and of apoptotic neutrophils by human Mf^12^, thus linking glucocorticoid action to phagocytosis and inflammation resolution processes^13,14^. Furthermore, a recent cellular and proteomic study reported that dexamethasone enhances Mo differentiation into Mf that can support erythropoiesis *in vitro* by phagocytosing extruded proerythrocyte nuclei^15^. Altogether this indicates that healthy human circulating blood Mo are physiologically relevant glucocorticoid target cells. Mo and the *in vitro* derived Mf are non-proliferating, non-transformed cells that represent an experimentally amenable primary human cell system to investigate glucocorticoid-induced epigenomic signalling in relation to cellular chromosome architectural features such as topologically associating domains (TADs).

Ligand-bound glucocorticoid receptor (GR, a.k.a NR3C1) is a transcription factor (TF) that belongs to the nuclear receptor superfamily^16,17^. GR-DNA crystals show that GR can interact in subtly different ways with different DNA sequences^18^ and that this is naturally modulated through alternative splicing of GR mRNA^19^. Chromosomal GR binding sites have been determined by chromatin immunoprecipitation (ChIP) coupled to next generation sequencing in several immortalised human and murine cell lines^19–24^, yielding several thousand binding sites. On the other hand, GR was reported to bind to only 338 genomic sites in primary human Mf ^25^. In mouse bone marrow-derived monocytes, about 1,300 GR ChIP-seq sites were observed, but nearly 8,000 new GR bound sites appeared upon stimulation with lipopolysaccharide (LPS), a cell wall component of gram negative bacteria^26^. Indeed, the epigenetic landscape has been proposed to play a determinant role in GR-mediated gene regulation by controlling DNA accessibility and potentiating GR chromatin binding in a cell type-specific fashion^23,27–30^.

The molecular mode of action of GR is still not fully understood^31^, in particular with regard to gene repression^32^. Transcription repression by DNA-bound GR has been suggested to occur through negatively acting GR DNA binding sites^33–36^. GR tethering to DNA by another DNA-bound transcription factor, as demonstrated by STAT3-dependent GR occupancy of sites lacking a canonical GR binding site, has been shown to correlate with a small number of glucocorticoid hormone-dependent transcription repression events in a mouse pituitary cell line^37^, and such mechanisms have been proposed for NFKB and AP-1 too as reviewed by Clark and Belvisi^32^. Still, ‘indirect’ repression via mutual inhibition of DNA binding with AP-1 components Jun and Fos was demonstrated as early as 1990^38–40^. Moreover, repression of IRF3 activity by GR can take place through competition for transcription co-activators such as mouse Ncoa2/Grip1/Src2/Tif2 which is rate limiting for both GR and IRF3 in immortalized mouse macrophages^41^, although the generality of the latter model has been called into question at the hand of *Tif2*^−/−^ mouse macrophages^35^.

To date, GR activity had not yet been compared side-by-side in primary human Mo and Mo-derived Mf. To this end we profiled the transcriptomes, histone H3-borne activating epigenetic marks and GR DNA binding sites after four hours of exposure to triamcinolone acetonide (TA) in human Mo and Mf. This revealed widespread modulation of the TA response by *in vitro* Mo to Mf differentiation. Combination of genome-wide data types (RNAseq, histone H3-ChIP, GR-ChIP) with human macrophage topologically associating domains (TADs)^42^, indicate that GR-induced epigenetic and transcriptomic signalling is significantly enriched in TADs bound by GR. Furthermore, transcriptomic and epigenetic signals induced by activated GR rarely if at all ‘spill over’ a TAD boundary. Our results are therefore consistent with the notion that TADs naturally integrate transcription regulation by distant *cis*-acting elements.

## Results

### Glucocorticoid-induced monocyte and macrophage transcriptome responses differ in magnitude

**Figure 1:**
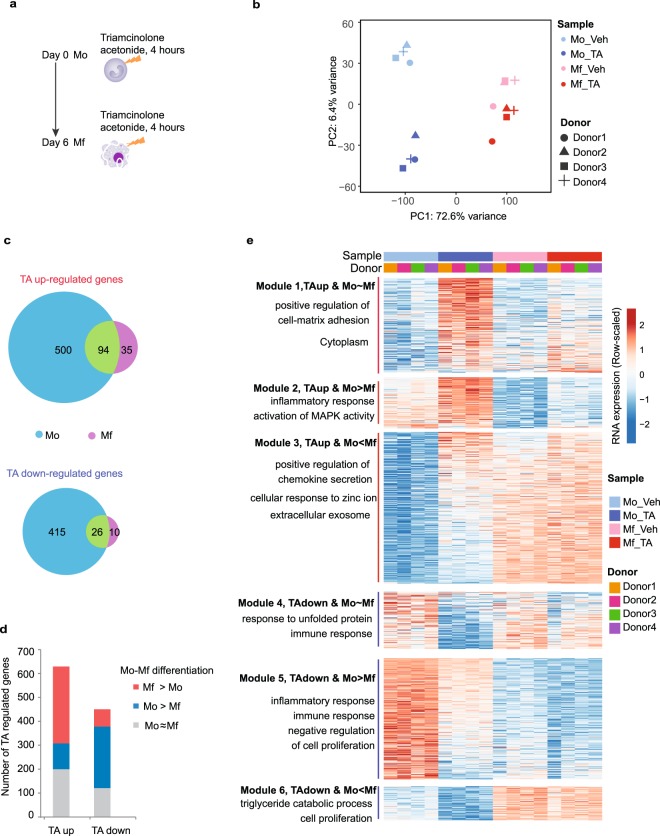
Transcriptomic impact of glucocorticoids on Mo and Mo-derived Mf. (**a**) Outline of the experiments performed in this study. Either freshly purified healthy donor monocytes (Mo) or the monocyte-derived macrophages obtained upon 6 days of *in vitro* differentiation (Mf) were exposed to 0.1% DMSO vehicle or 1 μM TA dissolved in DMSO for four hours. (**b**) Principal component analysis based on log2 normalized RNA-seq counts of the 5000 most variable genes using 16 samples derived from 4 donors. The shapes of the symbols indicate donors, colours indicate cell types and a darker shades indicates TA-treatment. (**c**) Venn diagram representations of the TA up- and TA down-regulated genes in Mo and in Mf. (**d**) Stratification of TA up- and down-regulated genes as a function of their relative activity in Mo and Mf. (**e**) Statistically significant transcripts up-regulated two-fold or more are plotted for each of the six combinatorial clusters shown in (**d**). Colouring is relative to the average of the 16 samples analysed in the four blood donors’ cells. Representative gene ontology biological processes are indicated for each cluster.

We profiled the transcriptomes of four healthy blood donors’ freshly purified monocytes (Mo) and of the respective *in vitro*-derived day six macrophages (Mf)^43^ upon a four-hour TA exposure (Fig. 1a, Table S1).

After batch effect removal (see materials and methods) principal component analysis reveals two principal components (PC) that explain 72.6% and 6.4% of the observed global transcriptome variance. PC1 reflects Mo to Mf differentiation and PC2 reflects TA exposure (Fig. 1b). Remarkably, the TA response is much stronger in Mo than in Mf, with 594 transcripts induced two-fold or more in Mo but only 129 in Mf, of which 94 are induced two-fold or more in both cell types (Figs 1c and S1a). Furthermore, TA led to reductions in 441 mRNAs in Mo but only 36 in Mf, with 26 responding in both cell types (Figs 1c and S1b).

To check the possibility that the TA dose of 1 μM was too high, we treated two additional healthy blood donor’s Mo with 100 nM TA or 100 nM dexamethasone and compared the resulting transcriptomes (Fig. S1c). The results indicate that, at the four hour time point, in most cases a marginally stronger response in terms of RNA level induction and suppression was attained using 1 μM TA, and all analysed genes showed very similar patterns of regulation by TA and dexamethasone (Fig. S1d).

To functionally validate the anti-inflammatory TA-response of the Mo-derived Mf, we stimulated three other donor’s Mf for 24 hours with LPS and measured cytokine release (Fig. S1e). As expected, LPS strongly induced IL6 and TNF secretion, and TA-treatment resulted in a dose-dependent inhibition of LPS-stimulated IL6 and TNF secretion (Fig. S1f,g). This was reversed by co-administration of the GR antagonist RU486 (Fig. S1f,g), verifying that TA regulates Mf pro-inflammatory signalling in our experimental set-up.

### Glucocorticoids impact the monocyte to macrophage differentiation expression program

The Mo and Mf TA responses differ in magnitude but are not discordant since no genes are induced two-fold or more in Mo but reduced in Mf or *vice versa*. Interestingly, stratification of the responses as a function of Mo to Mf differentiation reveals that over two thirds of the TA-response concerns genes that are themselves also subject to modulation as Mo differentiate into Mf (Fig. 1d). Strikingly, half (51%) of the TA up-regulated genes are more expressed in Mf than Mo, while only 17% are more expressed in Mo than Mf (Fig. 1d). Likewise, more than half (57%) of the TA down-regulated genes are more expressed by Mo than Mf, while only 16% are expressed more in Mf than Mo (Fig. 1d).

The six expression modules defined by intersecting the TA-response with Mo to Mf differentiation-driven gene expression dynamics (Fig. 1d) are displayed on Fig. 1e (Supplementary Dataset 1). The majority of the genes in these clusters are weakly responsive or even unresponsive to TA in Mf (Fig. 1e).

Gene ontology analysis shows that many biological processes are impacted by glucocorticoids, including cell-matrix adhesion, MAP kinase signalling, metabolism and immune responses (Fig. 1e), similar to conclusions from microarray studies^14,44^. Globally, 70% (n = 758) of the TA response concerns 15% of the genes that are differentially expressed by Mo and Mo-derived Mf (n = 5,069)^43^, suggestive of integration of glucocorticoid signalling in the Mo to Mf differentiation program^14,15^ with a prevailing trend towards activation of Mf-specific and repression of Mo-specific gene sets.

### Glucocorticoid epigenomic responses in Mo and Mf are H3K27ac driven

Because transcription changes often reflect histone H3K4me1, H3K4me3 and/or H3K27ac dynamics at enhancers and promoters, we mapped these three epigenomic marks in two additional healthy blood donors’ cells. The two donors show Pearson correlation above 0.9 for all three epigenetic marks, indicating high reproducibility (Fig. S2). Together, the epigenetically marked regions comprise 422 Mbp bearing one or more of the three histone H3 modifications we assessed (Supplementary Dataset 2).

**Figure 2:**
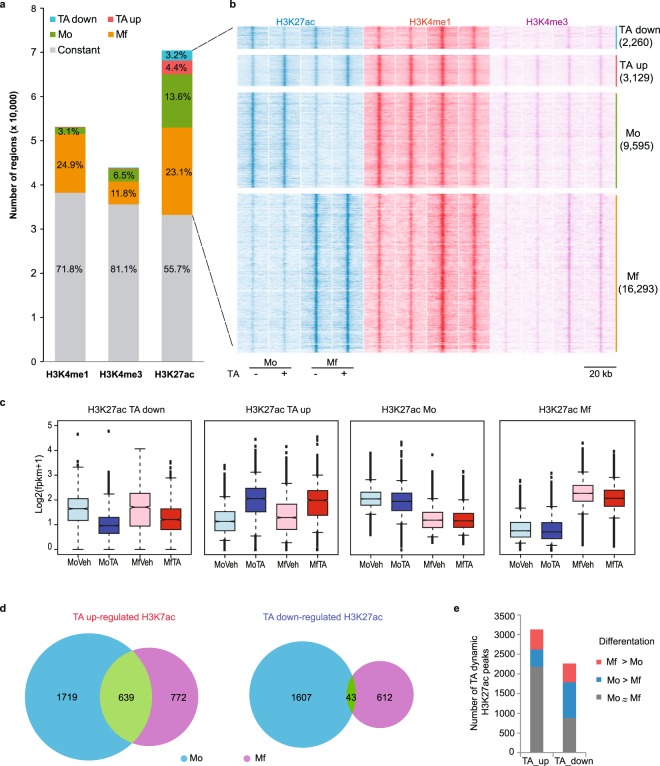
Genome-wide histone H3 modification pattern dynamics. (**a**) The absolute number of genomic regions decorated with H3K4me1, H3K4me3 and H3K27ac are plotted and coloured as a function of Mo-Mf differentiation and TA-treatment. See Supplementary Dataset 2 for the 123 H3K4me1 and 269 H3K4me3 regions that significantly respond to TA. (**b**) Fluff heatmap showing all the regions marked by dynamic H3K27ac patterns with respect to H3K27ac, H3K4me1 and H3K4me3 read counts. Numbers between brackets indicate absolute numbers of dynamic regions. (**c**) Boxplot representation of all the H3K27ac signal under the peaks called by MACS2 for the four sets of H3K27ac dynamic elements that are displayed in panel (b). (**d**) Venn diagram representation of the TA-induced and repressed H3K27ac-marked regions as a function of the cell type in which they were observed. Only effects in excess of 2-fold an with a DESeq2 P-value below 0.05 in both differentiation and TA treatment are shown. (**e**) Stratification of TA up- and down-regulated H3K27ac marking as a function of their relative activity in Mo and Mf.

There are 53,160 H3K4me1 regions that cover 14% of the human genome with a median width of 4,871 bp per region. Of these H3K4me1 regions, 28% (n = 14,894) are dynamic. When applying a lowered 1.5-fold change cut-off (P-value < 0.05), 89% of the H3K4me1 dynamic regions show increases in Mf relative to Mo. By the same criteria, only 0.2% of the H3K4me1 regions reacted to TA within 4 hours, with 117 increases and only six decreases (Fig. 2a). This marked H3K4me1 bias towards increases in the course of the 6 day *in vitro* cell culture period is identical to our past observation and broadly parallels shorter lived H3K27ac dynamics^43^.

H3K4me3 is embedded in H3K4me1 regions, marking gene promoters and certain active enhancers. It covers 2% of the genome with a median peak size of 693 bp. Of the 43,999 H3K4me3 peaks, 19% (n = 8318) are dynamic (2-fold, Pval < 0.05). Mo-Mf differentiation is associated with 99% of these H3K4me3 dynamic regions, of which about two thirds (n = 5,188) gain and about one third (n = 2,861) lose H3K4me3 signal in Mf relative to Mo^45^. There are 146 and 123 regions that respectively show significant gain or loss of H3K4me3 signal upon TA treatment (Fig. 2a). Of these 269 TA-impacted H3K4me3 regions, 25 are at promoter CpG islands, 77 at other types of promoters, and 167 map to distal enhancers.

Lastly, we detected 70,517 H3K27ac peaks with a median size of 574 bp that cover ~2% of the genome and that largely coincide with both H3K4me3 and H3K4me1 regions (Fig. 2a,b). Of all the H3K27ac-marked regions, 45% are dynamic as Mo differentiation proceeds and 7.6% (n = 5,389) respond to TA (Fig. 2b).

This epigenomic analysis demonstrates that in primary human Mo and Mo-derived Mf glucocorticoid signalling impacts histone H3K27 acetylation much more often than H3K4 methylation. This observation is in keeping with the fact that protein acetyl transferases are known to be recruited by GR^21,46–58^.

### Glucocorticoid-induced H3K27ac responses target Mo-Mf differential genes through distinct sets of enhancers

Quantification of H3K27ac dynamics (Fig. 2c) reveals that TA-induced H3K27ac responses are only slightly stronger in Mo than Mf, failing to explain the large difference in RNA level modulation between Mo and Mf. However, charting the H3K27ac regions as a function of their induction by TA (FC > 2, pval < 0.05) in Mo and/or Mf reveals that 55% (n = 1,719) are only significantly induced in Mo, 25% (n = 772) only in Mf, and 20% (n = 639) are significantly induced in both Mo and Mf (Fig. 2d). Hence, although the H3K27ac dynamics instigated by TA treatment are concordant between Mo and Mf, 80% are subject to modulation as Mo-Mf differentiation proceeds. Strikingly, for repressed H3K27ac regions, the significant response common to Mo and Mf cells only represent 2% (n = 43, Fig. 2d) of the observed dynamics, indicating a very strong epigenetic influence on the negative H3K27ac response elicited by TA. Accordingly, 61% of TA-repression occur at regions that change during Mo-Mf differentiation independently of glucocorticoid stimulation (Fig. 2e). By contrast, the majority (70%, n = 2,186) of TA-induced H3K27ac regions do not change significantly during Mo to Mf differentiation (Fig. 2e). Hence, a smaller proportion of TA-induced than TA-repressed *cis*-acting elements is subject to direct modulation in the course of Mo to Mf differentiation.

### GR protein levels are regulated by triamcinolone acetonide in primary human monocytes

**Figure 3:**
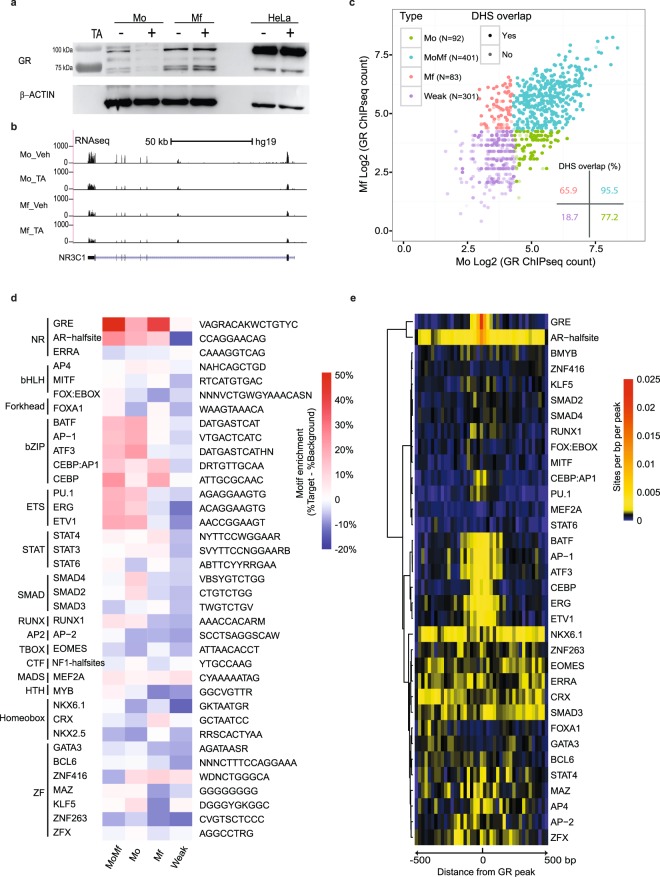
Genome-wide GR localisation patterns and underlying TF motif analysis. (**a**) Glucocorticoid receptor (GR) protein levels in Mo and Mf, treated or not with 1 μM TA for 4 hours. Beta-actin was used as internal loading control antigen. (**b**) RNA-seq signal at the *NR3C1* gene, which codes for GR, in Mo and Mf exposed for four hours to TA. (**c**) GR ChIP-seq signal intensity in Mo and Mf at 877 high confidence GR ChIP-seq peaks was used to define four sets of GR peaks (MoMf, Mo, Mf and ‘Weak’). Overlap with a previously determined set of Mo and Mf DHS^43^ is indicated by darker shading. The inset indicates the percentage of peaks that overlap with these DHS. (**d**) Enriched motifs in the four types of GR-bound regions sorted as a function of the TF superfamily they relate to. Colouring is based on the fractional difference between background and target frequencies. (**e**) Motif localisation density in the 401 MoMf-GR peaks, depicted as 40 bins of 20 bp.

Western blot analysis reproducibly indicated that untreated Mo show lower levels of GR protein than Mf (Fig. 3a). Furthermore, a reduction of GR protein occurs after four hours of Mo treatment with TA (Fig. 3a). By contrast, TA has no apparent effect on GR protein levels in Mf nor in HeLa cells (Fig. 3a). At the mRNA level, we find that Mo and Mf express the alpha isoform of GR^59^. In accord with GR protein levels, the GR transcript is reduced to 60% (padj = 1.6 × 10^−7^) in Mo upon four hours of TA-treatment (Fig. 3b). Surprisingly, GR mRNA is lowered in Mf to 35% of the level it shows in Mo (padj = 7.50 × 10^−27^), even though Mf display higher GR protein levels (Fig. 3a). These results indicate differential modulation of GR activity at the post-transcriptional level in the course of *in vitro* Mo differentiation.

### Glucocorticoid receptor chromatin localization in Mo and Mf

Since we profiled transcriptomes and epigenomes after four hours of TA stimulation we employed the same time point to profile GR genomic binding in four new healthy donors’ cells in the absence and presence of TA. Two samples were used to determine GR binding sites in Mo and two in Mf (Supplementary Dataset 3). In all ChIP-seq experiments TA clearly induced GR chromosomal occupancy (Fig. S3a), but the four GR ChIP-seq experiments differed in their dynamic range (Fig. S3b), reflecting differences between individual samples.

MACS2 analyses yields 877 GR peaks (P-value < 10^−7^), of which 401 are strongly bound in both Mo and Mf, while 92 are strongly bound only in Mo and 83 only in Mf (Fig. 3c). Furthermore, the MACS2 algorithm identified a set of 301 weakly bound genomic sites (Fig. 3c). Overall this number of GR binding sites is in keeping with a published experiment in human bone-marrow derived macrophages that identified 338 GR peaks^25^. The major fraction (45–52%) of the GR-bound sites is intergenic, 27–40% are intronic, 12–17% are in gene promoters and up to 8% are located in exons (Fig. S3c).

In line with previous observations in immortalised cell lines indicating that GR is preferentially localised to DNaseI hypersentive sites (DHS)^23,28–30^, 95.5% of the 401 strong GR peaks detected in both Mo and Mf occur in the set of DHS we have previously determined for Mo and Mf^43,60^, while 65.9% and 77.2% of the Mo and Mf-specific GR-bound sites and only 18.7% of the 301 weakly bound sites are located in such a DHS (Fig. 3c inset).

The 877 high confidence GR binding sites are generally spaced along the length of chromosomes by more than 10^6^ bp, similar to results obtained in HeLa and A549 cancer cells^21,22^, although a minority of binding sites (n = 45) occur within 30 kb of a second site (Fig. S3d). The latter map to 30 genes that are up-regulated in both Mo and Mf and includes well-known GR target genes *FKBP5*, *ZBTB16*, *TNFAIP3*, *PDK4* and *TSC22D3*, as well as clusters of GR target genes such as the Metallothionein cluster members *MT2A*, *MT1X*, *MT1F* and *MT1E* at chr16p13.3, *DUSP1* and *ERGIC1* at chr15q35.1, *SRGAP2D* and *FAM72D* at chr1q21.1, *SLC35E3*, Carboxypeptidase M (*CPM*) and *MDM2* at chr12q15 and *ADORA3* and *C1orf162* at chr1p13.2.

Intersection of the GR ChIP-seq binding sites with TA-up-regulated epigenomic signalling, indicates that the 401 sites common to Mo and Mf display local (within 500 bp) induction of H3K27ac (Fig. S4a). Mo and Mf cell type-specific GR-bound sites display TA-up-regulated H3K27ac signal in the corresponding cell types (Fig. S4a). Finally, the large majority of the 301 weak GR peaks did not display any local dynamics as little H3K4me1 or H3K27ac signal was present, even though H3K4me3 can locally mark such weak chromosomal GR binding sites (Fig. S4b,c).

We conclude that certain strongly responsive loci harbour multiple occupied GR binding sites that stimulate the expression of GR target genes known to be activated by glucocorticoids in many cell types. Nevertheless, most TA-responsive loci harbour only one strongly bound GR site that can act from a great distance to modulate promoter activity, as can be observed for the adjacent *FURIN*, *FES* and *MAN2A2* genes at chr15q26.1 which are all apparently induced by a single GR peak located about 100 kb away in the seventh intron of the *BLM* gene (Fig. S5a). This analysis indicates that the most common signature of TA-induced gene expression is having one or more strong GR peaks that overlap open chromatin (DHS) and a gain in H3K27ac signal.

### Transcription factor motifs at GR binding sites in Mo and Mf

There are several modes of responsiveness to TA in Mo and Mf (Fig. 1e). This may be due to differential TF activity at GR’s genomic target sites. Hence, to find out which TFs may be responsible, we scanned the DHS that overlap with our GR ChIP peaks with a set of 320 ChIP-seq TF motifs obtained from the Homer database^27^. This analysis points out 37 enriched TF motifs that are present in at least 5% of the scanned DHS (Fig. 3d).

Glucocorticoid response elements (GRE, GRACANNNTGTYC, Fig. S3e) are found in 79% of the 401 MoMf GR peaks, 48% of the Mo peaks, 34% of the Mf peaks and only 11% of the weak GR peaks. Steroid receptor half sites, best represented by the Homer androgen receptor-half site (CCNGGNACA, Fig. S3e), are present in 80, 73, 70 and 40% of the MoMf, Mo, Mf and Weak GR bound sites, respectively. Altogether 5, 24, 22 and 56% respectively, appear to lack both GREs and AR half sites. This indicates that GR binding was best detected at full steroid response sites and that a substantial fraction of the GR-bound sites that lack a canonical GRE does harbour steroid response element half sites (Fig. S3e), in line with results obtained in mouse liver and mouse bone marrow-derived Mf^31,61^.

In terms of potential ancillary TFs for GR, our analysis captures bZIP and ETS TF motifs in both the 401 MoMf and the 92 Mo-specific GR peaks. Motifs for SMAD TFs - acting downstream of TGFb signalling - are only enriched in Mo-specific GR peaks. On the other hand, Mf-specific GR peaks harbour more STAT motifs that function downstream of cytokine and growth factors that act through Janus kinases. The bZIP CCAAT/enhancer-binding protein (CEBP) motif is enriched in Mf and MoMf GR peaks (Fig. 3d). As described by others^61,62^, the bZIP and ETS motifs tend to be located within 150 bp of the GRE and therefore within one same nucleosome-sized DNA region as the GRE (Fig. 3e). Other enriched TF motifs such as the FOXA1 motif did not cluster as near to the GREs (Fig. 3e).

Altogether, 57 dynamically expressed TFs potentially correspond to the 37 enriched motifs that were significantly enriched at GR-bound regions. The RNA levels for dynamic members of each family (BCL6, bHLH, bZIP, C2H2, ETS, Forkhead, GATA, MAD, MADS, MYB, RUNX and STAT) are plotted in Fig. S5b. Overall, these results are consistent with the notion that lower expression of some of these TFs in Mf may in part explain the much lower GR-mediated transcriptional response observed in Mf as compared to Mo.

### Transcription factor expression modulation by glucocorticoids

Mining the RNA profiles revealed that 46 and 9 TFs are repressed by TA in Mo and Mf, respectively, of which 8 are shared. We recouped GR binding sites, epigenetic features and gene expression data using topologically associated domains (see below). This did not reveal convincing instances of repressed TF genes and associated chromosomal GR binding events. By contrast, upon recouping GR-binding sites and TA-induced epigenetic features, out of 39 induced TFs, we could identify 13 putative direct GR target TF genes as *ETS2, FOS*, *JDP2*, *CEBPD*, *KLF4*, *KLF7*, *KLF9*, *FOXN2* and *FOXO3*, *TFCP2L1*, thyroid hormone receptor beta (*THRB*), *ZBTB16*, the transcriptional repressor *TSC22D3* (a.k.a. *GILZ*) and the circadian transcription repressor *PER1*, where GR-bound putative enhancer to transcription start site distance ranges from less than 10 kb up to more than 100 kb as can be seen for *CEBPD* and *FOXN2* (Fig. S5c,d).

We conclude that in addition to the *TSC22D3*^63^, *PER1* and *ZBTB16* repressors, GR very likely directly activates bZIP, nuclear receptors, KLF, Forkhead and CP2 TF family members through binding to putative enhancers located up to several hundred kb from their promoters. Intriguingly, often there are multiple H3K27ac regions within these loci that show increased signal upon TA-stimulation (Fig. S5a,c,d).

### Induced but not repressed H3K27ac peaks often bear steroid response element motif instances

A striking observation in our data sets is that there are more induced H3K27ac peaks (n = 3,129) than GR binding sites (n = 877), of which 86% are associated with significantly induced H3K27ac levels.

The apparent relative excess of induced H3K27ac may indicate undetected GR binding events. To address this we scanned Mo and Mf DHS^43^ associated with all the H3K27ac peaks for the highly related glucocorticoid, mineralocorticoid, androgen and progesterone steroid response elements (Fig. S3e). This motif search establishes that 32% (n = 876) of the 2,719 TA-induced H3K27ac regions that are not associated with a GR ChIP-seq peak, do harbour a *bona fide* steroid response element. When scanning DHS adjacent to the 2,260 TA-repressed H3K27ac peaks (Fig. 2b), a smaller proportion (11%, n = 253) bear steroid response element motif instances, while 18% (n = 11,899) of 65,021 TA-insensitive H3K27ac peaks harbour steroid response elements. Hence, these motifs are enriched at induced relative to TA-repressed and TA-insensitive H3K27ac peaks (Fig. S4e) and undetected GR binding to GREs could account for an additional 876 TA-induced H3K27ac peaks. Nevertheless, only a minority of all accessible steroid response element motifs are occupied to the extent we could detect it experimentally.

Altogether, the motif scanning analysis displayed on Fig. S4e leaves 61% (n = 1892) of the H3K27ac-gaining regions that cannot be assigned to a GR ChIP-seq peak nor imputed as GR targets by virtue of being adjacent to a DHS bearing steroid response element motif instances. These induced H3K27ac peaks may correspond to secondary effects *in cis*, mediated by GR bound to neighbouring chromosomal loci. In order to test this hypothesis, we undertook it to map our data to topologically associated domains (TADs) since TADs are potential gene regulatory units within which long-distance communication between *cis*-acting elements is facilitated^64–68^.

### Transcriptomic and epigenomic characterisation of macrophage HiC TADs

**Figure 4:**
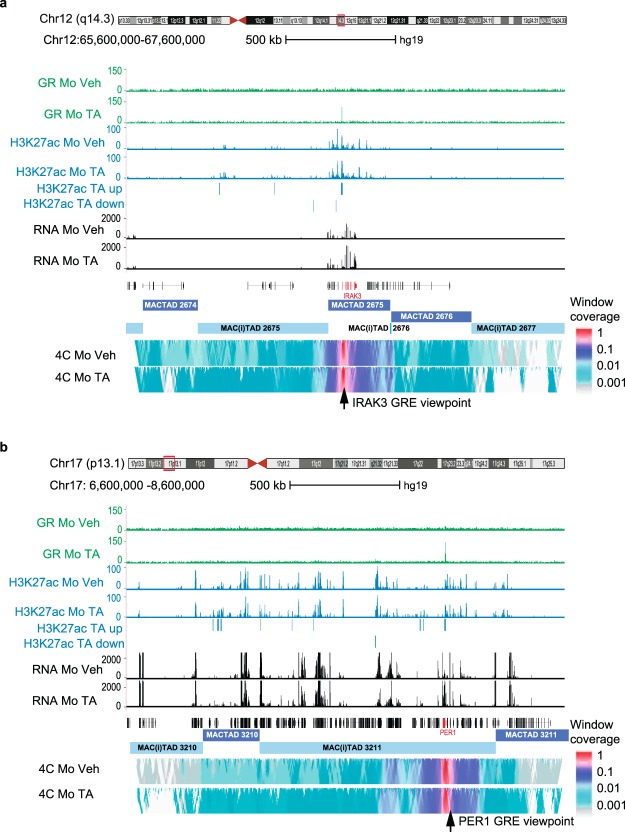
Circularised chromosome conformation capture using GRE viewpoints. (**a**) UCSC genome browser screen shot of a 2 Mb segment of chromosome 12q14.3 displaying; in green GR ChIP-seq results in Mo in the absence and in the presence of 1 μM TA, in blue H3K27ac occupancy, and in black RNA-seq data. UCSC transcripts are displayed (*IRAK3* is highlighted in red) above the MACTADs and MAC(i)TADs^42^. The two lower panels display median contact intensities of the *IRAK3* promoter GRE viewpoint with DNA in the 2 Mb window. Colouring is according to a normalised logarithmic scale^89^ in windows of increasing size (2 kb–50 kb from top to bottom). Note the asymmetry of the signal distribution relative to the viewpoint, which spans a region that rather accurately delineates MACTAD 2675 computed by Javierre *et al*.^42^. (**b**) As (a) but for the GRE located 2 kb upstream of the *PER1* gene transcription start site.

We performed circularised chromosome conformation capture (4C-seq) experiments in Mo at two of GR’s strongly bound target genes using the anti-inflammatory *IRAK3* and the circadian clock *PER1* genes’ GREs as 4C viewpoints^69–71^. Both GREs roam regions of approximately 250 kb (Fig. 4a,b). Comparison with a published set of 3,740 HiC human macrophage TADs (MACTADs)^42^ indicates that the relative median window 4 C coverage at *IRAK3* at chr12q14.3 outlines MACTAD 2675 rather accurately (Fig. 4a) while the *PER1* GRE appears to roam through approximately one third of the 1 Mb region located between MACTADs 3210 and 3211 (Fig. 4b). In both cases TA-treatment did not detectably affect the interaction frequencies of the GREs or the dimensions of the regions which are frequently in contact with the GREs. This confirms and extends previous studies on other GR target genes in immortalized mouse and human cell lines^63,72–75^.

**Figure 5:**
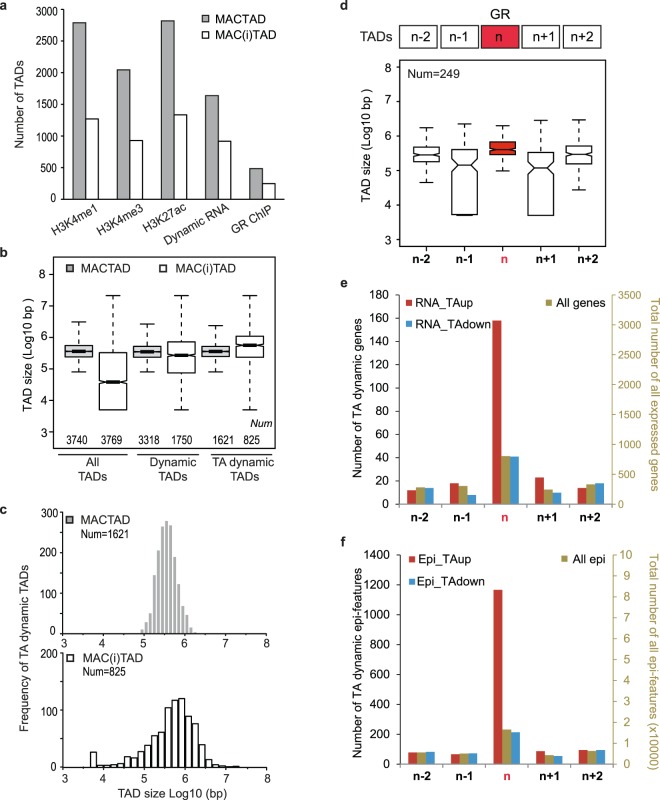
Adjacent TAD analyses. (**a**) Total number of MACTADs and MAC(i)TADs harbouring dynamic epigenetic regions, RNAs and GR peaks. (**b**) The first pair of box plots show the size distribution of all 3470 MACTADs delineated by Javierre *et al*.^42^ and of the intervening 3769 MAC(i)TADs, including MAC(i)TADs abutting centromeres and telomeres. The middle pair of box plots concerns TADs that harbour any dynamic feature as displayed in panel (a), and the third pair of box plots only concerns TADs bearing TA-induced features. (**c**) Histogram of the size distribution of the 1621 MACTADs and of 825 MAC(i)TADs bearing TA-responding features. (**d**) Schematic representation and size-distributions of 249 sets of four TADs that do not harbour any GR peaks themselves and that flank 249 TADs bearing at least one of the 401 MoMf-GR peaks depicted in Fig. 3b. (**e**) Total number of RNAs (right y-axis) and of TA-up-, and TA-down-regulated mRNAs (left y-axis) in the GR-bearing TADs and in the 249 sets of four neighbouring TADs. (**f**) Total number of epigenetic features (right y-axis) and of TA-up-, and TA-down-regulated histone H3-bearing epigenetic features (left y-axis) plotted as for (**e**).

Altogether, the 3,740 MACTADs leave 41% of the human genome unaccounted, as is the case of the *PER1* gene, which is not assigned to a MACTAD (Fig. 4b). Hence, to be able to chart the entire human genome, we initially called all the regions located between the MACTADs ‘inter-TADs’ (MAC(i)TADs). Including telomere and centromere coordinates results in 3,769 MAC(i)TADs plus 23 centromere and 46 telomere regions (we left out the Y chromosome as not all our blood donors possess it). The genetic content of MACTADs and MAC(i)TADs is rather similar, with respectively 29,043 and 24,489 human transcription start sites^76^. Next, we mapped our ChIP and RNA-seq data to MACTADs and MAC(i)TADs. This reveals that Mo and Mf epigenomic and transcriptomic features are found about twice more often in MACTADs than MAC(i)TADs (Fig. 5a). Although the average and median sizes of MACTADs and MAC(i)TADs are similar, especially when focussing on TADs that bear TA-responsive features (Figs 5b,c and S6a–d), MAC(i)TAD sizes show a larger spread than MACTADs, including 1,450 MAC(i)TADs that are between 5 and 10 kb long (Fig. S6b). The very small MAC(i)TADs such as MAC(i)TAD 2676 (Fig. 4a), likely represent ‘interstitial TADs’, while larger MAC(i)TADs such as MAC(i)TAD 3211 (Fig. 4b) may represent multiple TADs that still need to be resolved. We conclude that MAC(i)TADs harbour many relevant transcriptomic and epigenomic Mo-Mf features and henceforth refer to both MACTADs and the intervening MAC(i)TADs simply as TADs.

### GR-bound TADs concentrate up-regulated epigenomic features

Next, we attributed TA-up-regulated H3K27ac peaks to TADs with a GR chromosomal binding event. There are 733 TADs with at least one GR ChIP-seq peak shown in Fig. 3c. These TADs harbour 51% of the 3,129 TA-up-regulated H3K27ac peaks displayed on Fig. 2b (hypergeometric P-value 4.9 × 10^−136^). Inclusion of the 331 TADs that harbour at least one of the 876 up-regulated H3K27ac peaks associated with a DHS with a GRE motif but no GR peak (Fig. S4e), accounts for 81% of the 3,129 up-regulated H3K27ac peaks. This indicates that putative *cis-*acting elements showing TA-induced H3K27ac marking tend to reside in TADs that are decorated with multiple induced H3K27ac peaks and GR.

A conservative TAD-based analysis, using only the 401 strong GR peaks detected in both Mo and Mf (Fig. 3c), pinpoints 340 TADs of which 270 harbour 1,328 induced H3K27ac regions (hypergeometric P-value 1.5 × 10^−166^). Of these 1,328 regions, 326 are associated with a GR-peak and 260 only with a GRE. Hence, 742 TA-induced H3K27ac regions with neither a GR peak nor a GRE are located in TADs where they are potentially responding *in cis* to regulatory DNA modules that are bound by GR.

We conclude that TA-induced H3K27ac peaks that we could not ascribe directly to GR by ChIP-seq nor by GRE motif scans are often located in TADs that are bound by GR elsewhere, suggesting frequent interactions between GR-bound *cis-*acting elements and other *cis-*acting elements located in the same TAD.

### GR-instigated epigenetic signalling conforms to TAD dimensions

Next, we reasoned that if TADs indeed represent regulatory units to which epigenomic interactions are restricted^66,77^, GR-bound TADs would harbour more TA-instigated epigenetic and transcriptomic signals than the adjacent left and right neighbour TADs. To test this, we counted the number of TA-up-regulated and TA-down-regulated transcripts and epigenetic features in the four TADs that flank 249 non-adjacent TADs bearing one or more of the 401 MoMf-GR peaks (Figs 3c and 5d). We choose to use the two left and right neighbouring TADs because GR peaks are often located in well-defined MACTADs that are flanked by tiny interstitial TADs (see above, Fig. 5d), that could mask long range GR effects that would span across TAD boundaries.

Adjacent TAD analysis reveals a strong enrichment of up-regulated but not of down-regulated transcripts (Fig. 5e and S6e) and an even stronger enrichment of TA-up-regulated, but not of TA-down-regulated, epigenetic features within the GR-containing TADs when compared to adjacent TADs (Figs 5f and S6f). As control we counted all the expressed genes and epigenetic features, regardless of their (non)dynamic behaviour. In this regard the GR-bound TADs are not different from their neighbours (right-hand axis on Fig. 5e,f).

To estimate statistical significance, for each of the MoMf-GR-bearing TAD and the four flanking TADs we normalized the number of induced and repressed transcripts and the number of induced and repressed epigenetic features by the total number of expressed genes and the total number of epigenetic features, respectively. We performed a binomial test with null probability of 1 in 5 for the MoMf-GR-bearing TAD to have a higher ratio of induced or repressed features than the other four adjacent TADs. The real distribution for induced epigenetic features is 134/249 (pval = 4.39 × 10^−32^) and for induced transcripts it is 100/249 (pval = 2.64 × 10^−13^). For repressed epigenetic features and transcripts, we obtained respectively real distributions of 28/249 (pval = 0.99) and 27/249 (pval = 0, 99). Hence also at the TAD level we do not find statistical support for DNA-bound GR-mediated epigenomic gene repression.

The above results suggest that TADs can be used to computationally integrate signalling by GR-bound *cis*-acting elements because GR-induced signalling appears to largely respect the set of human macrophage HiC TAD boundaries defined by Javierre *et al*.^42^.

### TAD-based hierarchical clustering of RNA and ChIP data sets

**Figure 6:**
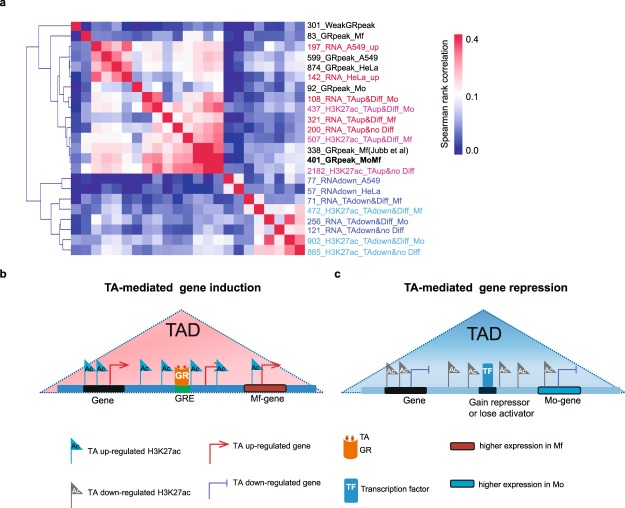
Correlation heat map of RNA partitions, GR and epigenetic marks based on assignment to TADs. (**a**) The data presented in Figs 1–3 and RNA-seq and GR-ChIP data for HeLa^21^, and for A549^22^ cells were assigned to TADs. Pairwise Spearman correlations were calculated and used to hierarchically cluster the data types. The colour scale renders Spearman correlation values. Red and blue text labels indicate respectively, up- and down-regulation by TA. Black text indicates GR ChIP-seq data sets. Numbers report the absolute number of features encompassed by each data set. (**b**) Schematic model of gene induction by TA in Mo. The right-most gene is representative of cluster 3 (Fig. 1e). (**c**) As (**b**) but for TA-repressed genes. The left-most gene is repressed by TA in both Mo and Mf. The more common expression pattern concerns genes more expressed in Mo. The right-most gene is representative of cluster 5 (Fig. 1e).

In order to integrate our data sets in a hypothesis-free fashion at the level of TADs, we computed pair-wise Spearman rank correlation coefficients for the frequency of GR-binding, dynamic mRNAs and epigenetic marks in TADs. As controls we included one published set of previously determined human Mf GR-ChIP sites^25^, and HeLa^21,75^ and A549^22^ GR-ChIP and RNAseq data sets (Fig. 6a).

Average Spearman rank correlation yields two correlating clusters; one for induced features and one for repressed features. Crucially, GR binding sites cluster with induced gene expression and epigenetic marking, but not repression. Furthermore, the TAD-based approach tightly clusters HeLa and A549 cell GR-bound sites and glucocorticoid-induced mRNAs but not repressed mRNAs from these cell types. Nevertheless, glucocorticoid-repressed genes do occur in TADs harbouring H3K27ac regions that are repressed upon TA administration, but these do not co-occur with GR binding.

It therefore appears that TADs can be exploited as genomic units for epigenomic and transcriptomic long-distance regulatory interactions such as those displayed by GR-bound enhancers.

## Discussion

We compared the impact of synthetic glucocorticoids on the transcriptomes and epigenomes of purified primary human Mo and six day-old *in vitro* differentiated Mo-derived Mf^43^. Remarkably, TA-treatment significantly altered 1035 RNAs above 2-fold in Mo but only 165 genes in Mf. By the same criteria, A549 and HeLa cells respectively alter the expression of 274 and 199 genes in response to glucocorticoids^21,22^. We speculate that the manipulations Mo underwent as they were purified away from other cells present in the blood donation and the subsequent adaption to the Petri-dish environment that accompany our first four-hour TA treatment underlies a part of the extensive Mo TA responses we observe, since the Mo transcriptome is in flux during this period. Along the same line of thought, the low response of Mf cultured *in vitro* for 6 days may be explained by a lack of stimulation of molecular signaling pathways that are dynamic in the body but not *in vitro*, such as adrenergic signaling^43^. Strikingly, many genes induced by TA only in Mo reach a higher level in Mf that is no longer inducible by TA (Fig. 6b, cluster 3 on Fig. 1e). Similarly, many genes repressed by TA in Mo, appear to have reached a low level in Mo-derived Mf that is not further reduced by TA (Fig. 6c, cluster 5 on Fig. 1e).

As discussed in the results section, there are multiple signal transduction pathways and attendant TF expression dynamics that may explain the weaker TA response observed in Mf despite similar DNA occupancy levels by GR in Mo and Mf upon TA treatment (Fig. 3c). Furthermore, transcription co-regulators have been proposed to modulate GR activity^46–58,78,79^ and many chromatin remodelling factors are expressed less in Mf (Fig. S7), potentially underlying the dichotomy between Mo and Mf in terms of the magnitude of their TA response.

To our surprise, we were unable to identify negatively acting GR-bound sites (nGREs^32,33^) that instigate H3K27ac level reductions. Our data therefore do not support mechanisms of persistent ‘on-DNA’ GR-mediated gene repression, be it through indirect GR tethering to DNA by other sequence-specific transcription factors or via nGRE binding^33–35^. It has been indicated that nGRE binding and transcription factor-mediated GR tethering are less efficient than direct GRE DNA binding^35^. Hence weak or indirect GR-DNA binding may have escaped our detection. Still, our collection of 301 weak GR binding sites does not correlate with reductions in H3K27ac nor with mRNA transcription repression, either indicating that the nGRE and GR tethering models do not apply to human Mo and Mf or that our ChIP assays could not detect these particular types of GR-chromosome binding events. In addition, as we only used one time point for the GR-ChIP experiments our data cannot formally exclude the possibility that GR did bind transiently to DHS flanking repressed H3K27ac sites at an earlier time point. Finally, it may be that GR represses gene expression via local increases in H3K9 or –K27 trimethylation marks, which we did not test here. However, to methylate these lysines, their acetylation marks would have to be removed, which we then would have expected to result in lowered H3K27ac marking.

There are several plausible – indirect – scenarios for losses of H3K27ac instigated by activated GR (Fig. 6c). Firstly, almost 50 TFs display reduced mRNA levels upon 4 h of TA treatment (Fig. S5), including NFKB and AP-1 subunits^38^, which can explain local losses of H3K27 acetylation simply by a reduced level of gene activation by these TF. Secondly, altered activity of transcription co-factors that remodel nucleosomes and/or modify transcription factors and the RNA polymerase II machinery can also explain local TA-induced loss of H3K27 acetylation^14,26,61^. Thirdly, H3K27ac loss could be a secondary consequence of increased repressor activity such as *TSC22D3* (a.k.a. *GILZ*)^80^ which is itself a direct transcriptional target of GR^63^.

Lowering mRNA levels may also be a post-transcriptional event. Indeed, there is accumulating evidence for indirect ‘non-genomic’ glucocorticoid effects through transcriptional induction of RNA destabilising factors by GR. For instance, *ZFP36L2* codes for an RNA destabilising factor and it is induced by GR in Mo (this work) and also in keratinocytes^81^. Furthermore and rather intriguingly, mRNA decay mediated by direct GR binding to specific mRNAs, in particular *CCL2* and other neutrophil attracting factor mRNAs, could also explain transcript level decreases instigated by TA^82–86^.

One part of our analyses relied on disparate genomic data type integration at the hand of TADs. Computationally, the use of TADs is much simpler than proximity assignment using (sub-)megabase windows because it is not necessary to overlap systematically different genomic windows for each combination of features that is considered. This permits disparate genomic data type integration by platforms that implement a query language like the GMQL genomic data management system^87^. Clearly, the macrophage TAD set we used here is a first generation prototype that will benefit from refinement since it is solely based on one macrophage HiC data set^42^ and does not take into account CTCF motif orientation nor cohesin complex binding sites^66,88^. Nevertheless, as such this TAD set concentrated TA-induced peaks into TADs harbouring GR binding sites, and the TA-induced H3K27ac responses were confined to these TADs, as indicated by very significant statistical outcomes in adjacent TAD tests (Fig. 5). Our analyses are therefore not inconsistent with the theory that transcription units and the *cis*-acting elements that control them are confined along the length of chromosomes by TAD boundaries. The TAD-based integration strategy, which we illustrate here to search for positive and negative GR targets, could also be applied to refine TAD models^88^ by testing whether coinciding genetic, epigenomic and transcriptomic dynamics respect or violate newly proposed TAD boundaries.

## Methods and Materials

### Cell culture and stimulation

Sanquin blood bank donors gave written informed consent for epigenetic research (NVT0068.01). All methods were carried out in accordance with, and using protocols approved by, the Sanquin Ethical Advisory Council, as previously described^43^. Mo were differentiated into Mf in RPMI1640 supplemented with 10% charcoal-stripped human serum. Treatment with triamcinolone acetonide (TA, Sigma, T6501), dexamethasone (Sigma, D4902) or DMSO (solvent vehicle for the steroids) lasted 4 hours.

### ELISA assay

Cytokine production was determined using 96-well cell culture supernatants and commercial ELISA kits for TNF-a (R&D Systems) and IL-6 (Sanquin) following the instructions of the manufacturer. One hour before LPS treatment on day 6 of Mo differentiation^43^, the medium was refreshed and 1 nM to 1 μM TA and optionally 1 μM RU486 of the GR antagonist (Sigma, M8046) were added to the medium. LPS (100 ng ml^−1^) treatment lasted 24 h at which point the supernatant was collected for ELISA.

### RNA extraction

Total RNA was extracted using the QIAGEN RNeasy RNA kit with DNaseI on-column treatment. RNA quality control was checked with a Bioanalyzer 2100 (Agilent). Ribosomal RNA was removed using Ribo-Zero rRNA removal magnetic kits (Illumina). RNA fragmentation at 95 °C for 7.5 minutes yielded ~200 bp fragments. cDNA synthesis was performed using SuperScript III (Thermo Fisher). For quantitative RT-PCR pre-validation prior to library construction, cDNA was amplified using iQ SYBR Green Supermix (Bio-Rad) with primers for *GAPDH* and *FKBP5* (*GAPDH*, Forward: AGAAGGCTGGGGCTCATTTG, Reverse: AGGGGCCATCCACAGTCTTC; *FKBP5*, Forward: CGGCGACAGGTTCTCTACTTA, Reverse: ATCGGCGTTTCCTCACCATT).

### Western blotting

Cells underwent lysis on ice for 30 minutes in lysis buffer (50 mM Tris-HCl pH 7.6, 120 mM NaCl, 0.5% NP-40, 1 mM EDTA) supplemented with 1 × Protease Inhibitor Cocktail (Roche). Total protein was collected after centrifugation at 4 °C, 13,000 rpm for 15 min. Equal quantities were used for SDS-PAGE gel electrophoresis. Anti-GR antibody (Abcam Ab3579) and anti-beta actin (Ab8226, Abcam) were employed.

### Histone ChIP

Attached cells were fixed with 1% formaldehyde through gentle shaking at room temperature for 10 minutes and quenched with glycine. Fixed cells were incubated in 1% SDS lysis buffer (20 mM Hepes, 1% SDS, 1× PIC) at a concentration of ~15 million cells per ml, followed by sonication using Bioruptor (Diagenode) for 5 cycles (30 s ON; 30 s OFF) to achieve enrichment of ~200 bp DNA fragments. Chromatin quality was confirmed with a Bioanalyzer. Chromatin immunoprecipitation was performed using anti-H3K4me3 (Diagenode C15410003-50), anti-H3K4me1 (Diagenode C15410194) and anti-H3K27ac (Diagenode C15410196-10) on 33 µl chromatin (~500,000 cells) and antibody in 300 µl dilution buffer (0.01% SDS, 1% Triton X-100, 1.2 mM EDTA, 16.7 mM Tris, 167 mM NaCl) with overnight rotation at 4 °C. For each ChIP, 20 µl A/G magnetic beads were washed twice and then suspended in 20 µl dilution buffer with 0.5% BSA and 0.5% SDS. The bead suspension was added to the chromatin-antibody mix and rotated at 4 °C for 1 hour. Beads were washed once in low salt buffer (2 mM EDTA, 20 mM Tris, 1% Triton, 0.1% SDS and 150 mM NaCl), twice in high salt buffer (2 mM EDTA, 20 mM Tris, 1% Triton, 0.1% SDS and 500 mM NaCl) and twice in TE buffer (1 mM EDTA and 10 mM Tris). Chromatin was eluted using 200 µl elution buffer (0.1 M NaHCO_3_ and 1% SDS) at RT rotation for 20 min. To de-crosslink DNA, 8 µl 5M NaCl, 3 µl 10 mg/ml proteinase K were added followed by incubation at 65 °C for 4 hours with shaking. ChIP DNA was purified using the Qiaquick MinElute PCR purification kit. ChIP occupancy was checked by qPCR with *GAPDH*, *CRYAA* and *FKBP5* enhancer region primers (*GAPDH* Forward: CAGGTTTCCAGGAGTGCCTT, Reverse: ATTAGGGCAGACAATCCCGG; *CRYAA* Forward: GTGTCAGGTCTGATCCGCAT, Reverse: GTGGAGTGAACACACACCCA; *FKBP5* Forward: TCTGAATGTGGCTGGCACAT, Reverse: TGCTGTGCACTCTCTTTCCT).

### Glucocorticoid receptor ChIP

Attached cells were fixed with 1% methanol-free formaldehyde whilst shaking for 10 minutes followed by quenching with glycine. Fixed cells underwent lysis in PIC buffer (10 mM Tris, 10 mM NaCl, 0.2% NP-40, 0.2% Triton, 1 mM EDTA and were further incubated in buffer C (150 mM NaCl, 1 mM EDTA, 0.5 mM EGTA, 50 mM HEPES, pH 7.6). The lysed cells were suspended in 0.5% SDS incubation buffer (150 mM NaCl, 1% Triton X-100, 1 mM EDTA, 0.05 mM EGTA and 10 mM Tris) at a concentration of ~30 million cells per ml, followed by Bioruptor (Diagenode) sonication for 7 cycles (30 seconds on; 30 seconds off) to achieve enrichment of ~200 bp DNA fragments. Next, 100 µl sonicated chromatin (~3 million cells) plus anti-GR antibody (Diagenode C15200010-50), was incubated in 0.1% SDS incubation buffer (same as above) with 20 µl washed protein A/G beads with rotation at 4 °C overnight. Beads were washed 5 times; once with low salt buffer (2 mM EDTA, 20 mM Tris, 1% Triton, 0.1% SDS and 150 mM NaCl), twice with high salt buffer (2 mM EDTA, 20 mM Tris, 1% Triton, 0.1% SDS and 500 mM NaCl) and twice with TE (1 mM EDTA and 10 mM Tris). ChIP DNA elution, decrosslinking and downstream processing was performed as for histone ChIP.

### 4Cseq

Circularised chromosome conformation capture (4C) assays were performed using a modified published approach^75,89^. Briefly, 10 million attached cells were fixed with 1% final formaldehyde for 10 minutes, and quenched with glycine. The cells were lysed in the 30 ml lysis buffer (50 mM Tris pH 7.5, 150 mM NaCl, 0.5% NP-40, 1% Triton, 5 mM EDTA and 1X protease inhibitor cocktail (Roche) with rotation at 4 °C for 30 min. Isolated nuclei were then fully digested by DpnII followed by overnight ligation with 50 U T4 DNA ligase at 16 °C. Ligated circular DNA were purified after de-crosslinking incubation with proteinase K at 65 °C for 4 hours, followed by further digestion by NlaIII. The digested DNA was ligated again and purified. The 4C libraries were amplified with the viewpoint-specific primers with inverse PCR. For each viewpoint, 8 PCR reaction products were pooled to enhance the library complexity. The 4C PCR products were purified using Qiagen Quick PCR purification Kit. *PER1* viewpoint primers are AGTTGGGGTGGAGAGGATC (Forward) and TACCTCTTCCCTGGCAACATG (Reverse). *IRAK3* viewpoint primers are AAAGGCCTGCTTTACCAGATC (Forward) and GCCTCTGTTGCTCAGCATG (Reverse).

### Next generation sequencing

RNAseq, ChIPseq and 4Cseq library preparation was performed using the KAPA hyperprep kit (KAPA Biosystems) according to the manufacturer’s protocol. For end-repair and A-tailing, 5 ng DNA was incubated with end-repair and A-tailing buffer and enzyme and incubated first for 30 minutes at 20 °C and then for 30 minutes at 65 °C. Then adapters, DNA ligase and ligation buffer were added and incubated at 20 °C for 15 minutes. Post-ligation cleanup was performed using Agencourt AMPure XP beads, the bead to DNA ratio was 0.8, 88 µl beads were added to 110 µl ligated DNA by gentle pipetting and incubated for 10 minutes at room temperature. After an 80% ethanol wash, DNA was eluted and amplified using KAPA HiFi Hotstart ReadyMix and Primer Mix. For ChIPseq and RNAseq, the DNA was purified using the QIAquick MinElute PCR purification kit. The selected DNA fragments of ~300 bp size were collected using an Egel system. For 4Cseq libraries, DNA was purified using AMPure XP purification system with double size selection to selected DNA fragment of 300 to 1000 bp. DNA library fragment size was checked using a BioAnalyzer. qPCR was performed to verify library quality. Sequencing was performed on the Illumina NextSeq platform.

### Bioinformatic analyses of RNAseq

RNA sequencing reads quality was checked with FastQC and the reads were aligned to hg19 and Ensembl v72 human transcripts using bowtie^90^. Principal component analysis revealed that variability was introduced due to the use of single-end and of paired-end sequencing libraries. To remove this spurious batch effect, we applied the ComBat function of the R-package SVA^91^ on the log2 transformed normalized read counts and performed the final PCA analysis on the read counts corrected for this batch effect. Differential expression was assessed using DESeq2^92^ for three comparisons; TA effect in Mo, TA effect in Mf, and Mo to Mf differentiation (Supplementary Dataset 1). Importantly, in all these comparisons, the batch effect caused by the variability in the sequencing protocol (single-end vs paired-end) was explicitly modeled as a predictor. Thus differential analysis was performed on the original (non-corrected) raw sequencing counts. Differential genes were selected on the basis of a base mean expression level above 20 normalized reads for the 16 samples, a |log2FC| > 1 and padj < 0.05. Bigwig files were generated for UCSC genome browser visualization using Deeptools bamCoverage^93^.

### Bioinformatic analyses of Histone H3 ChIPseq

Sequence read quality was confirmed with FastQC and the reads were mapped to human genome hg19 using bwa^94^. Peak calling was performed by MACS2^95^ at P-value of 10^−8^ using size extension to 200 nt. H3K27ac and H3K4me3 regions were called with the default ‘narrow’ setting of MACS2. H3K4me1 peaks were called with the ‘broad’ setting. Individual histone modification peaks from different samples were merged in one peak file. The tag density per peak was counted from the bam files using Peakstats (https://github.com/simonvh/solexatools). DESeq2 was used for three comparisons, TA effect in Mo and in Mf, and Mo-Mf differentiation (Supplementary Dataset 2). For H3K4me1 dynamics, qualitatively identical similar results could be obtained using a 1.5 and a 2-fold change cut-off. However, as the median size of the H3K4me1 regions is almost 5 kb, this represents a median 25 nucleosomes per region. Hence, a 1.5-fold change represents a rather robust effect involving either all nucleosomes or a subset of the nucleosomes in that region but then undergoing a higher fold-change. Bigwig files were generated by Deeptools^93^ and used for UCSC genome browser visualization. Heatmaps of 20 kb windows were generated at the hand of the Fluff software package^96^.

### Bioinformatic analyses of GR ChIPseq

Sequence read quality was confirmed as for Histone ChIP. The matching donor’s untreated (DMSO solvent only) sample was used as background input for MACS2 peaks calling using a P-value cut-off of 10^−7^ and size extension to 200 bp. GR peaks were called in individual donor’s paired samples treated or not with TA for each experiment and then merged (Supplementary Dataset 3).

### Bioinformatic analyses of 4Cseq

The 4Cseq raw data was mapped to hg19 and analyzed with the perl program 4Cseqpipe^89^. Forward primers were used as reading primers.

### Gene ontology analysis

GO analysis was performed using the DAVID web tool^97^. Gene sets were analyzed for enriched GO terms (biological processes) using all human genes as the background.

### Bioinformatic analyses of TF Motifs

Transcription factor motif instances were identified using Homer software^27^. Motif enrichment was calculated as the difference between motif frequency in target and in a random background of Mo-Mf DHS sequences. The motif distribution near MoMf GR peaks was detected by Homer and plotted by pheatmap (http://CRAN.R-project.org/package=pheatmap).

### Bioinformatic analysis of topological domains

The integration of epigenetic and transcriptomics data at the level of topological domains and relative statistical analysis was performed using spreadsheet software and queried using the GMQL engine^87^.

### Data sets

Raw data and processed data can be accessed via Gene Expression Omnibus (GEO) accession no. GSE109440. A public UCSC genome browser session displaying all our data is available at: http://genome.ucsc.edu/s/Colin%20Logie/Wang_Logie_Mo_Mf_TA_2018.

## Supplementary information

Dataset 1

Dataset 2

Dataset 3

Supplementary Table and Figures
