# Supplementary material for: Extensive epigenomic integration of the glucocorticoid response in primary human monocytes and in vitro derived macrophages
Source: Sci Rep. 2019 Feb 26;9:2772. doi: 10.1038/s41598-019-39395-9 (PMC6391480; doi:10.1038/s41598-019-39395-9)
Supplement: Supplementary file 4 — Supplementary Table and Figures [file 41598_2019_39395_MOESM4_ESM.pdf]

Supplementary information: Extensive epigenomic integration of the glucocorticoid response in primary human monocytes and in vitro derived macrophages

Cheng Wang[1], Luca Nanni[2], Boris Novakovic[1,3], Wout Megchelenbrink[1], Tatyana Kuznetsova[1,4], Hendrik G. Stunnenberg[1], Stefano Ceri[2], and Colin Logie[1,*]

[1] Department of Molecular Biology, Radboud Institute for Molecular Life Sciences, Faculty of Science Radboud University, PO box 9101, 6500 HG Nijmegen, The Netherlands
[2] Department of Electronics, Information and Bioengineering (DEIB), Politecnico di Milano, Piazza Leonardo da Vinci 32 - 20133 Milano, Italy
[3] Present address: Murdoch Childrens Research Institute, Royal Children's Hospital, Parkville, Australia
[4] Present address: Department of Medical Biochemistry, Academic Medical Centre of the University of Amsterdam, The Netherlands

* corresponding author: c.logie@science.ru.nl

Buffycoat	RNA-seq	H3K4me1-ChIP	H3K4me3-ChIP	H3K27ac-ChIP	GR-ChIP
bc9	MoMf_TA				
bc10			MoMf_TA		
bc11		MoMf_TA		MoMf_TA	Mf_TA
bc12	MoMf_TA			Mo_TA	Mo_TA
bc16				Mf_TA	
bc19		MoMf_TA	MoMf_TA		
bc27	MoMf_TA				
bc28	MoMf_TA				
bc33					Mo_TA
bc45					Mf_TA
bc52	Mo_TA-DEX 100nM				
bc53	Mo_TA-DEX 100nM				

Table S1 Healthy adult blood donation buffy coats and the assays performed on them.

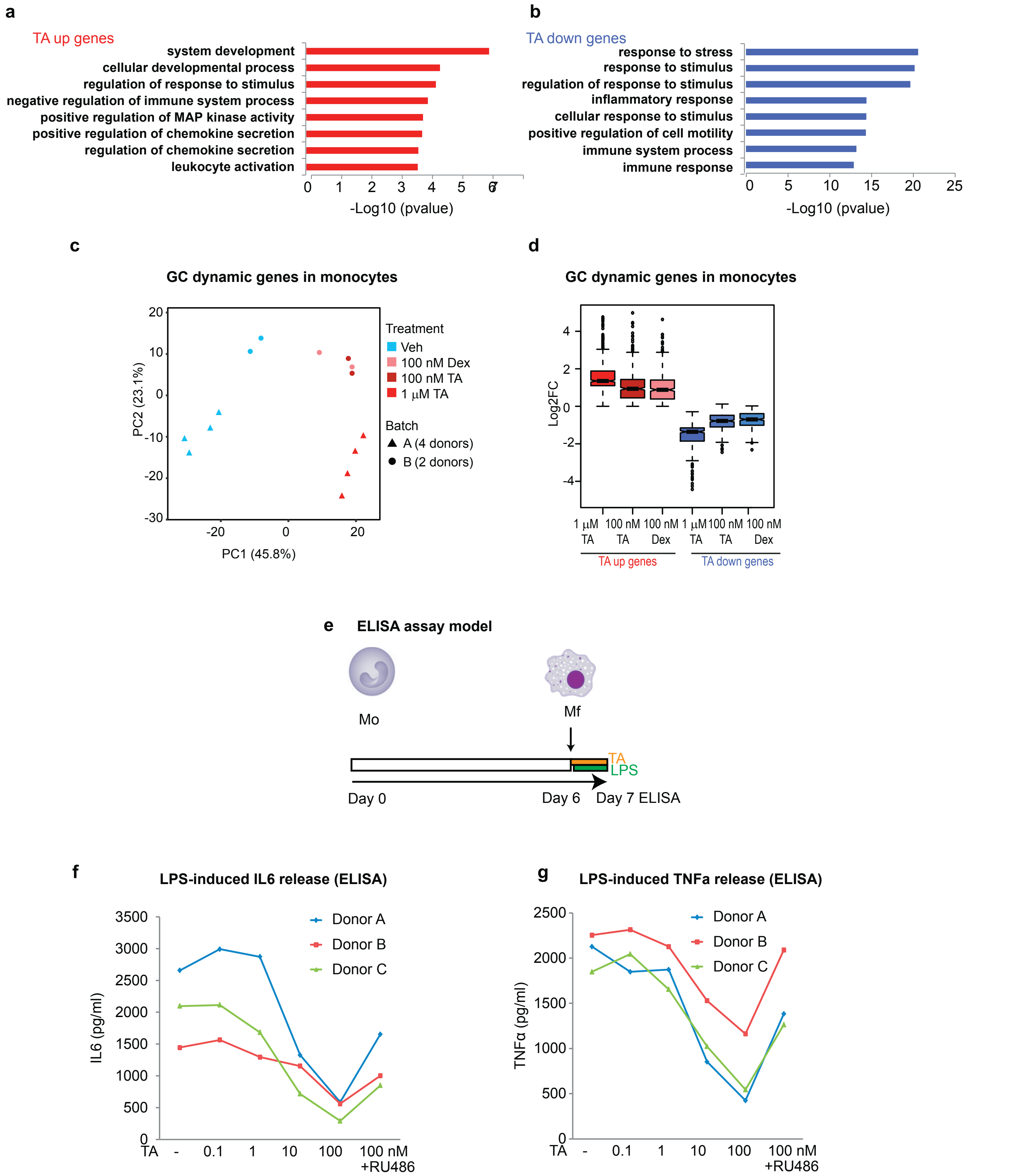

Figure S1

(**a**) Gene ontology analysis of the 629 TA up-regulated genes displayed on Fig. 1c-e.

(**b**) As (a) for the 451 down-regulated genes displayed on Fig. 1c-e.

(**c**) Principal component analysis of the eight samples derived from four donors' Mo exposed to 1 µM TA (triangles) displayed in Fig. 1b and of two additional donor's Mo samples exposed to vehicle (0.1% DMSO), 100 nM TA or 100 nM dexamethasone (circles). Symbol colour and shading indicate conditions.

(**d**) Box plot representation of the log2 fold-change for TA-dynamic gene in TA (1 and 0.1 µM) or dex (0.1 µM).

(**e**) Time line of LPS stimulation of Mo-derived Mf for the experiments displayed in panels (f) and (g).
TA was added 1 hour before LPS. The Elisa measurement was performed 24 h later.

(**f**) ELISA results against IL6 as a function of the indicated escalating TA doses in three donor's Mo-derived Mf.
RU486 was used at a concentration of 1 µM.

(**g**) As (f) but for TNF alpha release.

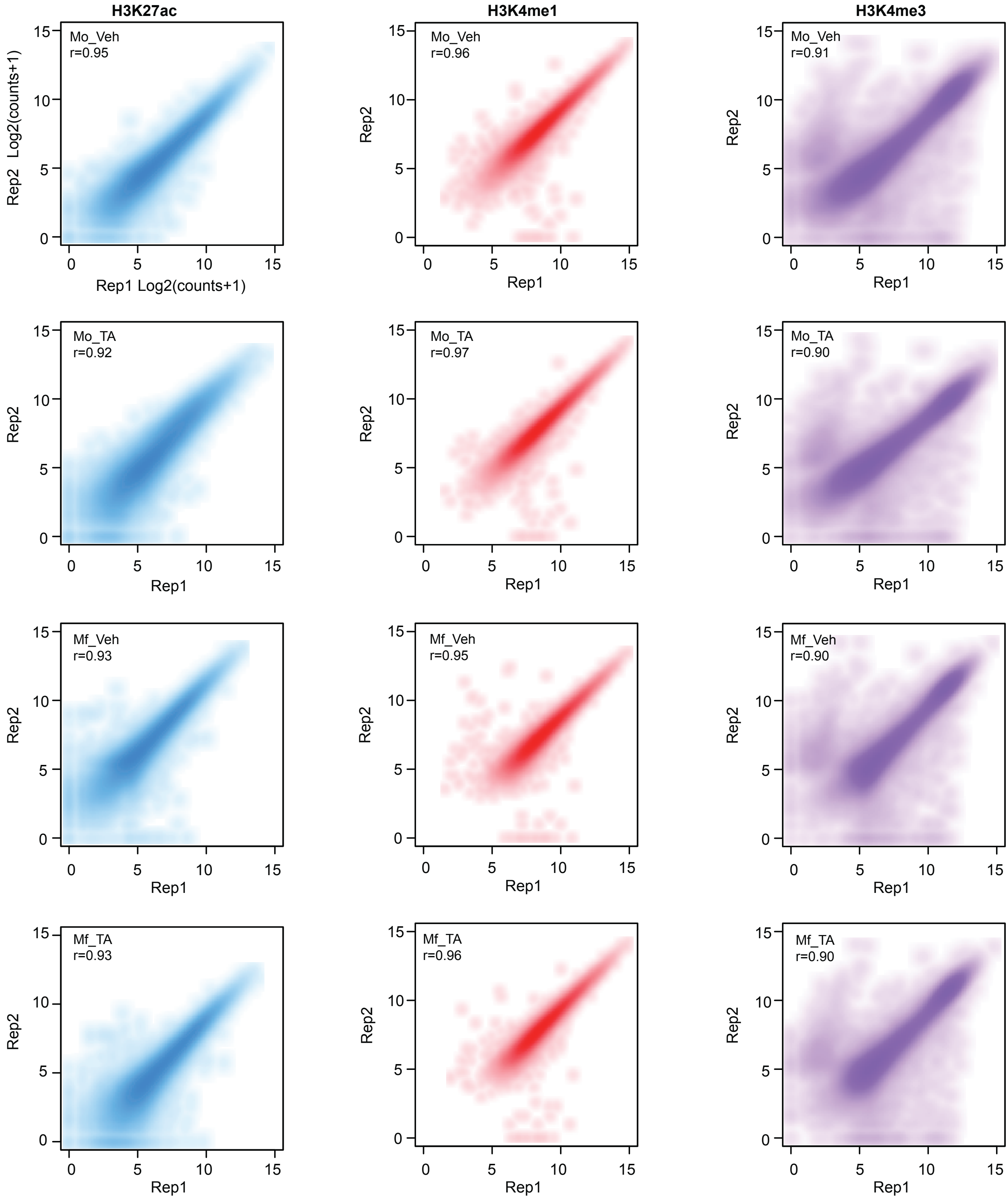

Figure S2

Pairwise comparisons of the post-translationally modified histone H3 ChIP-seq experiments performed with two healthy donors' cells. Note the Pearson correlation coefficients which are always above 0.9.

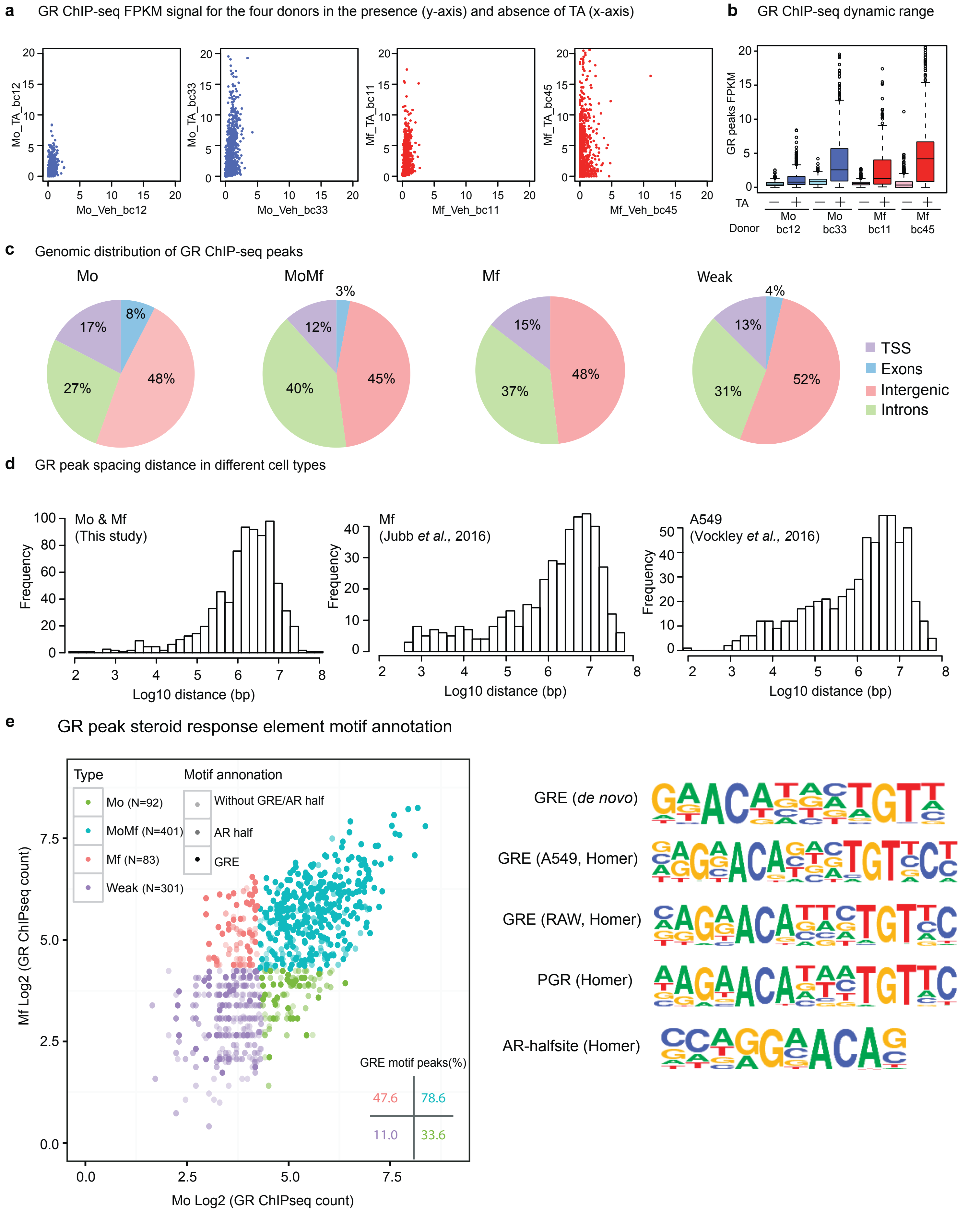

Figure S3

(**a**) Scatter plots of the four GR ChIP-seq experiment whereby the ChIP signal in the absence and presence of TA are plotted against each other for each donor sample pair. (**b**) The dynamic range of the four GR ChIP-seq experiments is indicated by the distributions of signal rendered as box plots. (**c**) Pie chart depiction of the proportions of GR-peaks in exons , introns, intergenic regions and transcription start sites for the four sets of GR peaks depicted in (e). (**d**) Distribution of intervals between GR peaks in three independent data sets. Since 23 chromosomes are considered, the 877 GR peaks (Fig. 3b) yield 854 intervals. The intervals between GR ChIP-seq peaks detected in bone marrow derived human macrophages (BMDM)[1] and the top 599 GR ChIP-seq peaks of A549 cells[2] are shown for comparison. (**e**) GR signal intensity in Mo and Mf at the 877 high confidence GR ChIP-seq peaks defines four sets of GR peaks (MoMf, Mo, Mf and 'Weak'). Overlap of each peak with bona fide AR half-sites and GREs is indicated by shading. The inset indicates percentages of peaks that harbour a GRE. GRE, PGR and AR half-site sequence logo's obtained from the Homer database and used to scan DHS associated with H3K27ac-marked regions are depicted next to panel e.

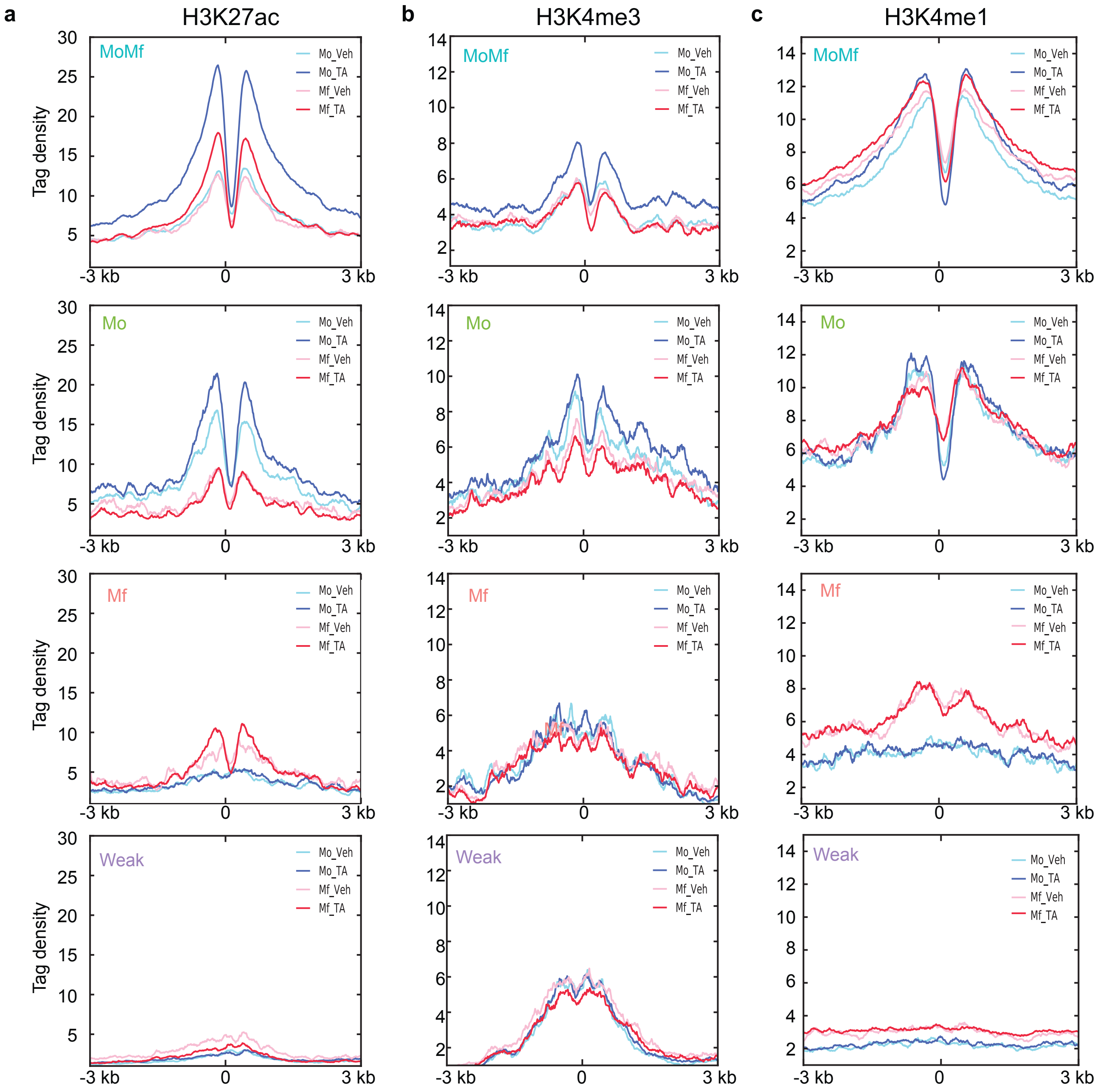

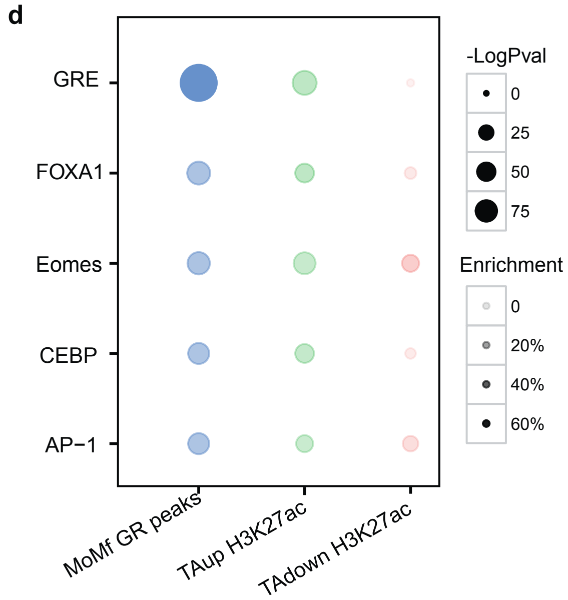

e

	TAup H3K27ac		TAdown H3K27ac		TA not affected		
	GR peak overlap		GR peak overlap		GR peak overlap		
	Yes	No	Yes	No	Yes	No	All H3K27ac
Steriod receptor binding motif	361	876	2	253	222	11899	13613
No Steriod receptor binding motif	49	1843	6	1999	112	70410	56797
All H3k27ac peaks	410	2719	8	2252	334	64687	70410

Figure S4

(**a**) H3K27ac ChIP-seq signal intensity in 6 kb windows centred on the four types of GR binding sites displayed in Figure 3c. (**b**) and (**c**) as (a) but for H3K4me3 and H3K4me1, respectively. (**d**) Statistical (P-value) enrichment (% above background) rendition of the indicated TF motif instances enriched in DHS bearing any one of the 401 MoMf GR peaks depicted on Fig. 3C in DHS associated with up- and down-regulated H3K27ac regions displayed on Fig. 2B. Note the depletion of lineage determining CEBP- and GR-associated motifs in TA-down-regulated H3K27ac DHS. (**e**) Contingency table of the occurrence of any of the full steroid receptor motifs (displayed on Fig. S3e) in H3K27ac-associated DHS that gain, lose or are not affected by TA for peaks that overlap one of the 877 GR-binding sites (Fig. 3c) or not, and arranged in two rows as a function of the presence or absence of steroid receptor motif instances.

Fig. S5

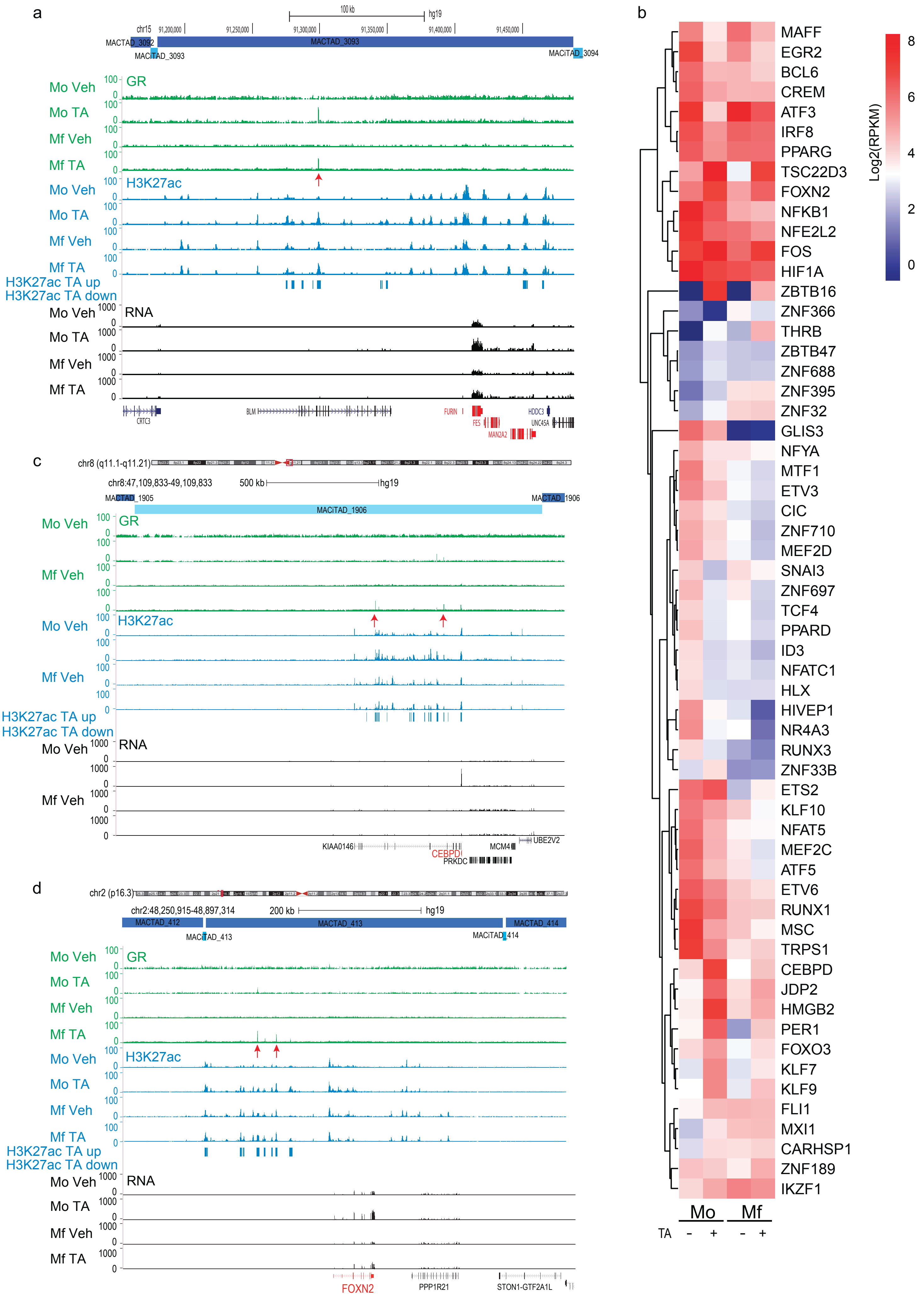
a
chr15
100 kb
hg19
MACTAD_3092
MACTAD_3093
MACiTAD_3093
MACiTAD_3094
GR
Mo Veh
Mo TA
Mf Veh
Mf TA
H3K27ac
H3K27ac TA up
H3K27ac TA down
RNA
CRTC3
BLM
FURIN
FES
MAN2A2
HDDC3
UNC45A
b
MAFF
EGR2
BCL6
CREM
ATF3
IRF8
PPARG
TSC22D3
FOXN2
NFKB1
NFE2L2
FOS
HIF1A
ZBTB16
ZNF366
THRB
ZBTB47
ZNF688
ZNF395
ZNF32
GLIS3
NFYA
MTF1
ETV3
CIC
ZNF710
MEF2D
SNAI3
ZNF697
TCF4
PPARD
ID3
NFATC1
HLX
HIVEP1
NR4A3
RUNX3
ZNF33B
ETS2
KLF10
NFAT5
MEF2C
ATF5
ETV6
RUNX1
MSC
TRPS1
CEBPD
JDP2
HMGB2
PER1
FOXO3
KLF7
KLF9
FLI1
MXI1
CARHSP1
ZNF189
IKZF1
Mo
Mf
TA
Log2(RPKM)
c
chr8 (q11.1-q11.21)
chr8:47,109,833-49,109,833
500 kb
MACTAD_1905
MACiTAD_1906
MACTAD_1906
KIAA0146
CEBPD
PRKDC
MCM4
UBE2V2
d
chr2 (p16.3)
chr2:48,250,915-48,897,314
200 kb
MACTAD_412
MACTAD_413
MACTAD_414
MACiTAD_413
MACiTAD_414
FOXN2
PPP1R21
STON1-GTF2A1L

Figure S5

(**a**) UCSC genome browser screenshot of chromosome 15q26.1 depicting MACTAD 3093 harbouring the TA-inducible genes *FURIN*, *FES* and *MAN2A2* genes and a single MoMf-GR ChIP-seq peak (red arrow) in the 7th intron of the *BLM* gene.

(**b**) Heat map clustered on the basis of expression patterns for TFs that change expression at least two-fold as a function of TA-exposure in Mo and/or Mf. The colour scale indicates the level of expression (log2 RPKM).

(**c**) UCSC genome browser screenshot of chromosome 8q11.1-q11.21 depicting MAC(i)TAD 1906 which bears the TA-inducible *CEBPD* gene that codes for the bZIP CCAAT enhancer binding protein D transcription factor. TAD dimensions, GR ChIP-seq signal (red arrow), H3K27ac ChIP-seq signal, called dynamic H3K27ac regions and RNA-seq signal are displayed above the human genes. Note the presence of at least twenty TA-induced regions that largely coincide with non-coding RNA KIAA0146 and the *CEBPD* gene in a region that extends 385 kb downstream from *CEBPD*.

(**d**) As for (c) but chromosome 9q31.2-q31.3 depicting MACTAD 2146 which bears the TA-inducible gene *FOXN2*, multiple GR binding sites (red arrows) and many induced H3K27ac regions.

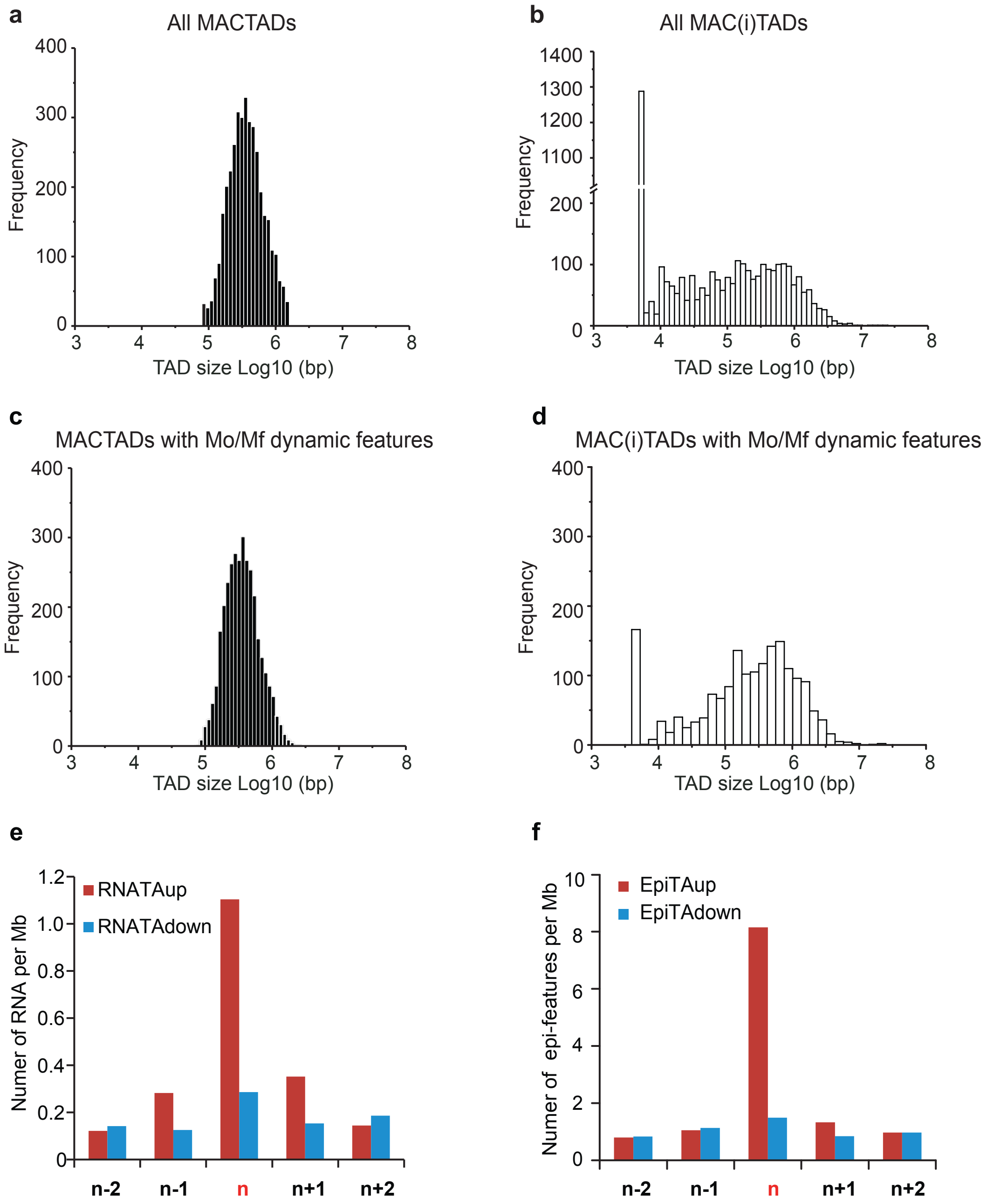

Figure S6

(**a**) Histogram of the size-distribution of all MACTADs (n=3,740). (**b**) Histogram of the size-distribution of all MAC(i)TADs (n= 3,769). (**c**) Histogram of the size-distribution of all MACTADs that bear GR, dynamic genes and/or epigenetic features (n=3,318). (**d**) Histogram of the size-distribution of all MAC(i)TADs that bear GR, dynamic genes and/or epigenetic features. (n=1,750). (**e**) Density (features per Mb) of TA-up-, and TA-down-regulated transcripts in 249 GR peak-bearing TADs and the associated sets of four neighbouring TADs. (**f**) As (e) but for H3-bourne epigenetic features.

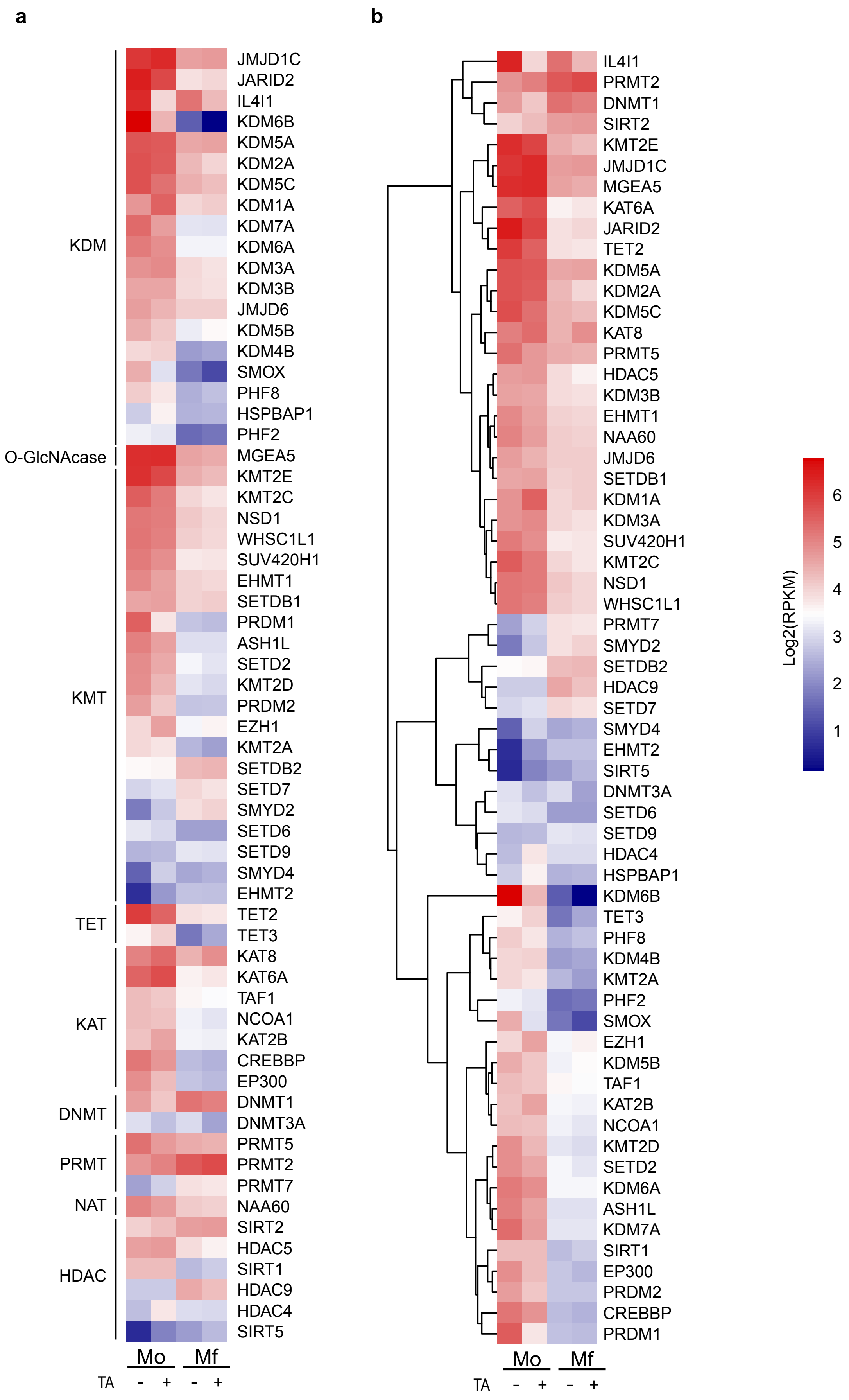

Figure S7

(**a**) Heat map clustered on the basis chromatin remodelling enzyme types ('writers' and 'erasers') that change expression at least two-fold as a function of Mo to Mf differentiation and/or TA-exposure. The colour scale indicates the level of expression (log2 RPKM). (**b**) As (a), but clustered on expression dynamics rather than on enzyme type.

References

1. Jubb, A. W., Young, R. S., Hume, D. A. & Bickmore, W. A. Enhancer Turnover Is Associated with a Divergent Transcriptional Response to Glucocorticoid in Mouse and Human Macrophages. *J Immunol* **196**, 813-822, doi:10.4049/jimmunol.1502009 (2016).

2. Vockley, C. M. et al. Direct GR Binding Sites Potentiate Clusters of TF Binding across the Human Genome. *Cell* **166**, 1269-1281 e1219, doi:10.1016/j.cell.2016.07.049 (2016).
